# Lack of Gender Disparities in Emergency Department Triage of Acute Stroke Patients

**DOI:** 10.5811/westjem.2014.11.23063

**Published:** 2014-12-01

**Authors:** Tracy E. Madsen, Esther K. Choo, Todd A. Seigel, Danielle Palms, Brian Silver

**Affiliations:** * Alpert Medical School of Brown University/Rhode Island Hospital, Department of Emergency Medicine, Providence, Rhode Island; † Kaiser Permanente East Bay, Department of Criticial Care Medicine, Department of Emergency Medicine, Oakland, California; ‡ Emory University, Department of Epidemiology, Rollins School of Public Health Atlanta, Georgia; § Alpert Medical School of Brown University/Rhode Island Hospital, Department of Neurology, Providence, Rhode Island

## Abstract

**Introduction:**

Previous literature has shown gender disparities in the care of acute ischemic stroke. Compared to men, women wait longer for brain imaging and are less likely to receive intravenous (IV) tissue plasminogen activator (tPA). Emergency department (ED) triage is an important step in the rapid assessment of stroke patients and is a possible contributor to disparities. It is unknown whether gender differences exist in ED triage of acute stroke patients. Our primary objective was to determine whether gender disparities exist in the triage of acute stroke patients as defined by Emergency Severity Index (ESI) levels and use of ED critical care beds.

**Methods:**

This was a retrospective, observational study of both ischemic and hemorrhagic stroke patients age ≥18 years presenting to a large, urban, academic ED within six hours of symptom onset between January 2010, and December 2012. Primary outcomes were triage to a non-critical ED bed and Emergency Severity Index (ESI) level. Primary outcome data were extracted from electronic medical records by a blinded data manager; secondary outcome data and covariates were abstracted by trained research assistants. We performed bivariate and multivariate analyses. Logistic regression was performed using age, race, insurance status, mode of and time to arrival, National Institutes of Health Stroke Scale, and presence of atypical symptoms as covariates.

**Results:**

There were 537 patients included in this study. Women were older (75.6 vs. 69.5, p<0.001), and more women had a history of atrial fibrillation (39.8% vs. 25.3%, p<0.001). Compared to 9.5% of men, 10.3% of women were triaged to a non-critical care ED bed (p=0.77); 92.1% of women were triaged as ESI 1 or 2 vs. 93.6% of men (p=0.53). After adjustment, gender was not associated with triage location or ESI level, though atypical symptoms were associated with higher odds of being triaged to a non-critical care bed (aOR 1.98, 95%CI [1.03 – 3.81]) and 3.04 times higher odds of being triaged as ESI 3 vs. ESI 1 or 2 (95% CI [1.36 – 6.82]).

**Conclusion:**

In a large, urban, academic ED at a primary stroke center, there were no gender differences in triage to critical care beds or ESI levels among acute stroke patients arriving within six hours of symptom onset. These findings suggest that ED triage protocols for stroke patients may be effective in minimizing gender disparities in care.

## INTRODUCTION

Gender disparities have been observed in the use of intravenous (IV) tissue plasminogen activator (tPA) for acute ischemic stroke, one of the few known treatments to improve long-term outcomes in this condition.^1^–^5^ In addition, women are less likely to meet quality markers for stroke: they are less likely to have non-contrast computed tomography (CT) within 25 minutes and to receive IV tPA within one hour.^3^,^6^

Factors leading to these disparities have never been identified, though provider bias may be a contributor. Specifically, emergency department (ED) triage, a critical decision point, provides an opportunity for unintentional provider bias because of its inherent subjectivity.^7^,^8^ In addition, ED triage affects time to provider evaluation in stroke patients^9^ and influences time to provider, ED length of stay, and mortality in other patient populations.^10^,^11^ Furthermore, ED triage level and location could serve as targets for interventions to improve ED care and decrease potential disparities.

During ED triage of a potential stroke patient, triage nurses assign both an acuity level and an ED bed. Though there is no uniformly adopted ED triage tool in the United States, the most commonly used system is the Emergency Severity Index (ESI), a 5-level scale used to triage ED patients from least acute (ESI 5) to most acute (ESI level 1).^7^,^8^,^12^,^13^ The ESI tool has been externally validated multiple times, but may be less accurate in certain demographics of patients, including the elderly.^7^,^13^,^14^ Bed assignment is the second decision of the triage process, as patients perceived as needing critical interventions may be transferred immediately to a resuscitation area. Placement in an ED critical care bed could be considered a surrogate measure of aggressiveness of care and affects time to provider in the study hospital’s ED.

ESI level is a predictor of wait times for patients and thus may contribute to delays in time to provider assessment of stroke patients, though ESI level has not yet been studied as a possible contributor to ED delays in women with stroke.^9^,^10^ The guidelines for use of the ESI tool suggest that patients with acute neurologic deficits be triaged as an ESI 1 or 2. Patients who require immediate intervention or resuscitation meet criteria for level 1, while those with a “high risk situation” or abnormal vital signs should be assigned an ESI level of 2. If patients do not meet the criteria to be an ESI 1 or 2, the number of anticipated resources is used to assign patients into ESI categories 3, 4, or 5.^7^,^8^,^13^ Of note, lack of recognition of “high risk situations” has been shown to be a common reason for ESI 2 patients being misclassified as ESI 3.^14^

Previous studies have not investigated ED triage level or triage location by gender in stroke patients, despite the potential to use these factors as a method to reduce disparities in stroke care. Using ESI level and assignment to ED critical care beds, the objective of our study was to investigate ED triage of stroke patients by gender as a potential contributor to disparities in care, as those triaged as less acute may be subsequently treated less aggressively. Our study hypotheses were that women would be triaged to lower acuity ESI levels and to non-critical care beds more often than men. Secondary objectives included performance of non-contrast CT within 25 minutes of arrival, IV tPA given within 60 minutes of arrival, survival to hospital discharge, and discharge destination in women compared to men.

## METHODS

### Study Design/Patient Population

This study was a retrospective cohort study of ischemic and hemorrhagic stroke patients at least 18 years old admitted to a large, urban, academic ED between January 2010, and December 2012. We included in the study patients with a discharge diagnosis of acute ischemic stroke or intracerebral hemorrhage if they arrived at the ED within six hours of symptom onset. This time window was chosen in order to select patients who were potentially eligible for intervention at our institution. We excluded patients if they met the following criteria: 1) if they were transferred from an outside hospital or 2) if the discharge diagnosis was subarachnoid hemorrhage. The study was approved by the hospital’s institutional review board.

Given the lack of prior literature on specific triage outcomes in stroke patients, this was designed as a hypothesis-generating study. We chose the study period to incorporate a time period during which stroke triage protocols were introduced into the study hospital’s ED. Specifically, code stroke activations were introduced during early 2010; these activations consist of blast pages to ED physicians and on-call neurologists upon the arrival of a potentially tPA eligible stroke patient. An additional type of activation, “neurology team,” was introduced in early 2012. The “neurology team” activation by triage nurses alerts ED physicians that a potential stroke patient has been placed into a room.

### Primary and Secondary Outcomes

The primary outcomes were the following: 1) ESI score, assigned by the ED triage nurse, and 2) ED triage assignment to either a critical care or urgent area bed. The study hospital’s ED consists of three sections designated as “urgent” pods and a 12-bed section designated as the “critical care” pod. Triage personnel assign patients that warrant immediate evaluation to critical care beds rather than urgent area beds; for example, stroke patients arriving within the tPA time window as determined by the triage nurse are assigned to critical care beds. The two primary triage outcomes were not combined because of the concern that predictors of ESI may not be the same as predictors of triage to a critical care bed.

Secondary outcome variables were the following: 1) performance of non-contrast CT within 25 minutes of arrival, 2) IV tPA given within 60 minutes, 3) survival to hospital discharge, and 4) discharge destination.

### Data Collection

We collected data in two ways. First, data on primary outcomes (ESI level, assigned bed) as well as demographic variables (gender, age, race, and ethnicity) were extracted from ED electronic medical records by a data manager blinded to the study hypothesis. Approximately 15% of these data were validated by research assistants (RAs) to ensure accuracy.

Second, data pertaining to secondary outcomes and study covariates were abstracted from patients’ ED and inpatient electronic medical records by three trained RAs using standardized data abstraction forms. These data included initial vital signs, National Institutes of Health Stroke Scale (NIHSS), mode of arrival, medical comorbidities, initial presenting complaint as documented by the triage nurse, time to CT, survival to discharge, and discharge destination. Presenting complaints were categorized as atypical as defined in previous studies and included non-neurologic symptoms (chest pain, shortness of breath, nausea, vomiting), change in level of consciousness, and “generalized” weakness when not associated with other typical symptoms.^15^–^17^ Symptoms including unilateral weakness, sensory changes, visual changes, headache, dizziness/vertigo, and ataxia/gait difficulty were considered typical. Multiple RAs reviewed approximately 10% of charts, and inter-rater reliability for secondary outcomes and covariates was calculated using Cohen’s kappa; kappa values ranged from 0.80 to 1.00. Missing NIHSS scores were estimated retrospectively by two study investigators (TM and BS) using the documented physical exam on admission, a previously validated method.^18^,^19^ The weighted kappa value for missing NIHSS scores was 0.87.

### Data Analysis

We used means, medians, and proportions to describe the sample as appropriate. For bivariate analyses, Pearson’s chi-square tests, Student’s t-tests, or Wilcoxon rank-sum tests were performed as appropriate. Primary outcomes were the two triage measures: 1) ESI level and 2) triage location (critical care vs. urgent area bed); gender was the independent variable. ESI level was recoded into a binary variable (ESI 1 or 2 vs. ESI 3) after analyzing the distribution of the variable: only 4.84% (n=26) were triaged as ESI 1 so those patients were combined with ESI 2 patients. No patients were assigned ESI 4 or 5.

We performed multivariate regression with each of our two primary outcomes (ESI and triage location). With regard to missing outcome data, 76 patients had missing ESI scores and were excluded from the ESI regression model while only one patient had a missing triage location. Potential covariates were chosen a priori based on prior studies of ED triage, time to CT, and time to provider evaluation in stroke patients.^6^,^8^,^9^,^15^,^20^ Because literature on ED triage of stroke patients was limited, studies of time to CT and time to provider evaluation were also used to guide covariate choice; this method was felt to be plausible because ED triage is an important contributor to both time to CT and provider evaluation.

For each model, the potential covariates were gender, age, race, ethnicity, insurance status, initial systolic blood pressure, mode of arrival, presence of atypical symptoms, time to hospital arrival, and NIHSS. We initially included all potential covariates; covariates with significant multicollinearity (ethnicity and systolic blood pressure) were then removed. Time to arrival in minutes, initially a continuous variable (range: 3 – 360, IQR: 43 – 160, mean: 115, median: 80), was transformed into a binary variable because of its non-normal distribution. We also transformed NIHSS because of a non-normal distribution (range: 0 – 38, IQR: 3 – 17, mean: 10, median: 7). The final model for both triage location and ESI included gender, age, race, insurance status, presence of atypical symptoms, NIHSS (transformed using square root), arrival within three hours, and mode of arrival. We tested model fit using Hosmer–Lemeshow goodness-of-fit testing.

The gender-stratified models for triage location had adequate model fit. Gender-stratified results for ESI, however, were not reported due to potential model instability; this instability may be a result of missing data or unmeasured predictors of ESI.

Models were checked for potential bias introduced by the relatively rare outcomes of ESI 3 and triage to non-critical care beds using the Firth method of logistic regression.^21^ By using an alternate estimating method to maximum likelihood estimation, the Firth approach reduces potential bias resulting from using small samples or rare events.^21^,^22^ This method did not significantly change model coefficients or p-values. For logistic regression models, adjusted odds ratios (aOR) with 95% confidence intervals were reported. Stata version 12.1 was used for all analyses.

## RESULTS

We included 537 patients in our analysis; 264 (49.2%) were women, 91 (17.0%) were non-white, and 42 (7.8%) were Hispanic (Table 1). Women were about six years older than men on average (75.6 vs. 69.5, p<0.001) and were more likely to have a history of atrial fibrillation (39.8% vs. 25.3%, p=0.002). Otherwise, baseline characteristics including NIHSS scores were similar between men and women. A slightly greater proportion of women had atypical symptoms; however, this was not statistically significant (47.2% vs. 42.8%, p=0.31).

### Triage Location

**Figure 1:**
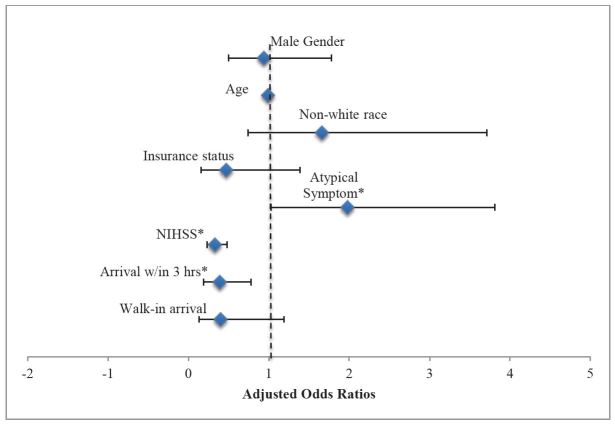
Adjusted odds of triage to non-critical care beds are displayed. *NIHSS,* National Institute of Health Stroke Scale Predictors are male gender, age, non-white race, insurance status, presence of atypical symptom (*p=0.04), NIHSS score as a continuous variable transformed using square root (*p<0.001), walk-in arrival compared to ambulance arrival, and arrival within 3 hours of symptom onset (*p=0.008). Model fit was tested using Hosmer–Lemeshow goodness-of-fit test, (χ^2^=7.87, p=0.45).

The majority of our sample was triaged to ED critical care beds (90.1%, n=483). Using bivariate analysis, there was no gender difference in the proportion of patients triaged to non-critical care beds (10.3%, women vs. 9.5%, men, p=0.77). In our multivariate model, after adjusting for age, race, insurance status, mode of arrival, atypical symptoms, NIHSS, and arrival within three hours, gender remained unassociated with triage to a non-critical care bed (aOR 0.94, 95% CI [0.50 to 1.78]) (Figure 1). Those with higher NIHSS were less likely to be triaged to non-critical care beds (aOR 0.33, 95% CI [0.23 – 0.48]), and those with any atypical symptoms were more likely to go to non-critical care beds (aOR 1.98, (95% CI [1.03 – 3.81]). Age was not significantly associated with triage location (aOR 0.99, 95% CI [0.97 – 1.02]).

After stratifying the sample by gender, non-white race was associated with triage to non-critical care beds in women but not men (aOR 4.80, 95% CI [1.63 –14.11], vs. aOR 0.44, 95% CI [0.10 – 1.89]). In addition, arrival within three hours was associated with triage to a non-critical care bed in women but not men (women, aOR 0.22, 95% CI [0.08 – 0.61] vs. men, aOR 0.56, 95% CI [0.19 – 1.68]), while presence of atypical symptoms was more strongly associated with triage location in men compared to women (men, aOR 2.82, 95% CI [1.08 – 7.43] vs. women, aOR 1.38, 95% CI [0.53 – 3.6]). For gender-stratified models, model fit was adequate for both women (χ^2^= 10.98, p=0.20) and men (χ^2^= 10.35, p=0.24). Lower NIHSS scores were associated with triage to non-critical care beds in both genders.

### ESI Level

**Figure 2:**
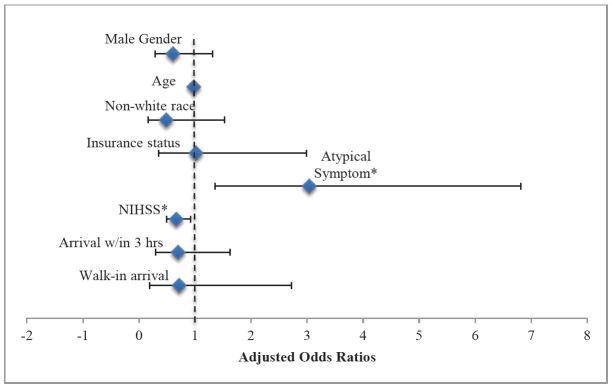
Adjusted odds of triage as ESI 3 compared to ESI 1 or 2 are displayed. *NIHSS*, National Institute of Health Stroke Scale Predictors are male gender, age, non-white race, insurance status, presence of atypical symptom (*p=0.007), NIHSS score as a continuous variable transformed using square root (*p=0.013), walk-in arrival compared to ambulance arrival, and arrival within 3 hours of symptom onset. Model fit was tested using Hosmer–Lemeshow goodness-of-fit test, (χ2= 15.7, p=0.05).

Most of the sample was assigned as ESI 2 upon triage (74.9%, n=402). Using bivariate analyses, there were no significant gender differences in the distribution of ESI levels between women and men (ESI 1: 4.6% women vs. 5.1% men, ESI 2: 74.6% women vs. 75.1% men, ESI 3: 6.8% women vs. 5.5% men, missing ESI: 14.0% women vs. 14.3% men, p=0.92). No patients were assigned triage levels of ESI 4 or 5. After adjusting for age, race, insurance status, presence of atypical symptoms, NIHSS, mode of arrival, and arrival within three hours, gender was not significantly associated with triage as ESI 3 versus ESI 1 or 2 (aOR 0.61, 95% CI [0.29 – 1.31]) (Figure 2). Of note, those categorized as having atypical symptoms were 3.04 times more likely to be triaged as ESI 3 versus ESI 1 or 2 (95% CI [1.36 – 6.82]). Age was not significantly associated with ESI level (aOR 0.98, 95% CI [0.95 – 1.00], p = 0.08). Model fit was adequate but had a borderline p-value (χ^2^= 15.7, p=0.05).

### Secondary Outcomes

Women and men were equally like to have non-contrast CTs within 25 minutes (45.8% vs. 46.9%, p=0.81). Similarly, of those with ischemic stroke who arrived within three hours of symptom onset, 39.2% of women compared to 40.8% of men received IV tPA within 60 minutes (p=0.84). At discharge, mortality between women and men was similar (11.4% vs. 11.4%, p=0.99). Women were, however, less likely to be discharged home from the hospital (33.5% vs. 47.0%, p=0.004).

## DISCUSSION

In our novel, exploratory study of potential triage disparities in acute stroke patients, there were no gender disparities in triage acuity levels or ED triage location. Though previous studies have reported gender differences in time to initial CT and time to provider evaluation,^6^,^20^ our study is the first to investigate ED triage as a potential contributor to these gender disparities. We found that overall, triage to non-critical care ED beds and assignment to less acute ESI levels were relatively rare events. Significant predictors of triage to non-critical care beds or less acute ESI levels included lower NIHSS scores and atypical symptoms. We found no significant association between age and triage status, and there did not appear to be gender-specific effects of age in our stratified models, despite the fact that women in our sample were, on average, older. Finally, we found a potential interaction between gender and race, with non-white women experiencing triage disparities in comparison to white women.

Our results are notable for several reasons. First, triage of acute stroke patients by gender has not previously been characterized. In our study, triage assignments did not differ significantly by gender among ED stroke patients. Given the lack of understanding around the contributors to gender disparities in treatment of acute stroke, this is an important finding. If this negative finding is confirmed in other hospital settings and in larger patient samples, attention should be shifted to other potential contributors to gender disparities in stroke treatment such as differences in tPA eligibility or subconscious physician bias.

Second, in contrast to prior studies, we did not find any gender differences with regard to time to CT or administration of IV tPA within 60 minutes.^6^ For example, in an analysis of a national stroke registry by Kelly et al.,^6^ women were less likely to have an initial CT performed within 25 minutes. Other literature has shown a gender disparity in time to physician evaluation.^20^ The differences between our findings and previous studies may be a result of different study populations; our sample was from a single academic stroke center, while the Kelly et al.^6^ study was a large, multi-center study including many community hospital EDs. We also must consider the possibility that the lack of gender differences in CT within 25 minutes and/or IV tPA within 60 minutes are the result of prompt and appropriate ED triage for both women and men, though this would require further investigation in a future study.

There are several potential explanations for the lack of gender disparities in our study. First, gender disparities in the care of acute stroke may be decreasing over time as the importance of time to treatment becomes more evident, and our data may reflect this.^23^ In addition, our findings suggest that the use of stroke triage protocols including nursing notifications of the arrival of acute stroke patients and a blast page system to indicate the arrival of tPA-eligible patients may decrease gender disparities in stroke care, though this will require investigation in future studies comparing EDs with stroke protocols to those without stroke protocols. Similarly, designated stroke centers could be compared to centers without such certifications with respect to potential gender differences in triage and/or stroke treatments. Previous literature regarding the effect of stroke center designation on treatment disparities is lacking. Though some preliminary data suggest that gender disparities in the use of IV tPA use are not eliminated at primary stroke centers (PSC),^24^ these particular data are limited by a very small absolute difference in tPA use between women and men (6.7% vs. 7.5%) as well as lack of adjustment for tPA eligibility, NIHSS, and changes in tPA use over time. Other previous literature suggests that gender and racial disparities in tPA use are decreasing over time, though these studies lack the ability to control for stroke center designation.^23^,^25^ Finally, EMS protocols could have affected our results. The vast majority of our sample arrived via EMS; if pre-hospital providers had already identified these patients as potential strokes, this could have significantly affected ED triage.

Our findings of the other predictors of less acute triage assignments suggest additional directions for future research in ED stroke care. For example, the gender-specific effects of atypical symptoms and arrival times on ED triage we describe need to be confirmed and explored in future studies. In addition, our concerning finding that non-white women are more often triaged to non-critical care beds will need to be re-examined in further investigations of the interaction of race and gender in treatment disparities. Previous literature has demonstrated that non-white women experience more treatment disparities than other demographic subgroups,^26^ but the differences in triage will require confirmation. We also found that patients with lower NIHSS scores or atypical symptoms of stroke were more likely to go to non-critical ED beds or have less acute ESI assignments. Because less acute ESI assignments and placement in non-critical care beds likely lead to delayed diagnostic assessments, these findings should be confirmed in future studies. Additionally, triage protocols should be modified to ensure that patients with minor strokes and atypical symptoms are triaged so that they receive immediate assessment.

## LIMITATIONS

Our study has several limitations. It is a retrospective, single-center study; because of this, generalizability may be limited. Our outcomes of triage to non-critical care beds and less acute ESI levels were also relatively rare. Because of the low outcome rates, results taken from our gender-stratified models must be interpreted with caution. We anticipated these limitations, however, and intended our study to be exploratory in nature. In addition, our ED is part of a large, academic, high stroke volume hospital certified as a PSC: Code stroke protocols may reduce triage disparities and make the results less generalizable to EDs without these protocols in place. Data on other factors that may influence the triage of stroke patients including the gender of the triage nurse were not collected. Finally, data regarding time between arrival and triage assessment as well as time to physician assessment were not available; we used time to initial CT as a surrogate measure of physician assessment.

## CONCLUSION

In a sample of acute stroke patients seen in a large, urban, academic ED within a primary stroke center, we observed no gender differences in ED triage level or ED triage location. In academic designated stroke centers, triage is not likely to be a major source of delays for stroke care in women. In future research, stroke triage in EDs with organized stroke care and/or designated as stroke centers should be compared to other EDs to evaluate the effectiveness of organized stroke care in reducing gender disparities.

## Figures and Tables

**Table 1:** Selected characteristics of participants, by gender.

Characteristic	Women (n= 264)	Men (n=273)	p-value
Age (mean)	75.6 (73.9–77.4)	69.5 (67.7–71.4)	p<0.001
Non-white race	16.3% (43)	17.7% (48)	p=0.66
Hispanic ethnicity	8.3% (22)	7.3% (20)	p=0.66
Uninsured	12.6% (33)	16.1% (44)	p=0.25
NIHSS (median, IQR)	8.0 (3–18)	7.0 (3–15)	p=0.23
Arrival within 3 hours	76.5% (202)	81.3% (222)	p=0.17
Discharge diagnosis			
Ischemic stroke	88.3% (233)	83.9% (220)	p=0.14
Intracerebral hemorrhage	11.7% (31)	16.1% (44)	
Mode of arrival			
EMS	92.1% (233)	88.5 (239)	p=0.17
Walk-in	7.9% (20)	11.5% (31)	
Hypertension	80.3% (212)	83.9% (229)	p=0.28
Diabetes mellitus	28.0% (74)	28.9% (79)	p=0.82
Atrial fibrillation	39.8% (97)	25.3% (64)	p<0.001
CT within 25 minutes	45.8% (107)	46.9% (105)	p=0.81
Discharge destination			
Home	33.5 (88)	47.0 (126)	p=0.004
Other	55.1 (145)	41.4 (111)	
Died prior to discharge	11.4 (30)	11.6 (31)	

*NIHSS*, National Institute of Health Stroke Scale; *EMS*, emergency medical services; *CT,* computed tomography
